# Abnormal Gut Microbiota Metabolism Specific for Liver Cirrhosis

**DOI:** 10.3389/fmicb.2018.03051

**Published:** 2018-12-10

**Authors:** Xiao Wei, Jiangtao Zhao, Xiaodong Jia, Xiangna Zhao, Huan Li, Weishi Lin, Ruo Feng, Jing Yuan

**Affiliations:** ^1^Centers for Disease Control and Prevention of PLA, Beijing, China; ^2^Department of Histology and Embryology, School of Basic Medical Sciences, Zhengzhou University, Zhengzhou, China; ^3^Comprehensive Liver Cancer Center, 302 Military Hospital, Beijing, China

**Keywords:** cirrhosis, gut, microbiota, metabolic network, ammonia

The gut microbiota has been neglected in precision medicine for a long time and is considered as the virtual endocrine organ of the human body (Evans et al., 2013; Clarke et al., 2014). The intestinal microbiome is made up of a diverse group of microorganisms, including bacteria, archaea, and eukaryotes, living together intimately. There are 300 to 1,000 different species living in the colon, which contains a densely-populated microbial ecosystem (Knight and Girling, 2003; Sears, 2005). However, 99% of the bacteria come from about 30 or 40 species (Knight and Girling, 2003; Sears, 2005). The human gastrointestinal microbiome plays an essential role in protecting the intestine, promoting nutrient absorption, resisting infection, and promoting the development of the immune system (Clarke et al., 2014). Gut microbiota can metabolize some xenobiotics such as phytochemicals, food toxicants, and drugs. The intestine-brain axis is a biochemical signaling pathway that includes the gut microbiota, central nervous system, and the neuroendocrine and neuroimmune systems. The gut microbiota can be considered as a large endocrine organ with the potential to produce nearly 100 kinds of products, which are absorbed into the blood and act on the distal organ. In the human body, the relationship between various microorganisms is mutually restricted, and the growth and decline of any one kind of microorganism may cause human diseases. There is ample evidence that complex metabolism and biochemical activities of intestinal microbiota are complementary with human physiology (Hooper et al., 2002; Turnbaugh et al., 2009; Qin et al., 2010). The gut microbiota operates as a whole and has a close relationship with human health and diseases (Qin et al., 2010). The intestinal microbiota has wide participation in cometabolism with the body and has remarkable influence on the metabolic phenotype of the host (Qin et al., 2012; Wei et al., 2016b).

The liver is an important metabolic organ in the human body and has multiple functions, such as detoxication, secretory protein synthesis, glycogen storage, and so on. The liver has an intimate relationship with the gut through the physiological structure of the gut-liver axis (Szabo, 2015). The pathological mark of liver cirrhosis is hepatic fibrosis and portal hypertension, resulting in gastrointestinal congestion, tissue edema, slow gastric peristalsis, and intestinal permeability increase. Meanwhile, impaired liver function leads to the weakened ability to scavenge metabolites and decreased defense ability in patients; the combined effect of multiple factors leads to intestinal microenvironment imbalance and dysfunction. Many studies have shown that the intestinal microbiota community associated with cirrhosis is disordered, with a marked loss of *Bacteroides* and significant increases in *Veillonella, Enterobacteriaceae*, and *Streptococcus*. In addition, a distinct enhancement was detected in gut microbiota genes involved in material transport and metabolism, whereas a significant loss of cell cycle-related genes was detected in cirrhosis patients (Chen et al., 2011; Wei et al., 2013; Qin et al., 2014). During disease development, the intestinal microbiota is constantly adjusting itself and regulating the body's metabolism. For a biological organism, most of the functional proteins are classified as carbohydrate metabolism and amino acid metabolism pathways, and changes in some low-abundance proteins (such as cell communication, cell motility) are usually not so significant and are not easily detected.

## Gut Microbiota Community Change In Cirrhotic Patients

In the past few years, cirrhosis-related gut microbiota characteristics have been researched from the perspective of metagenome, metaproteome, and metabolome (Wei et al., 2013, 2016a,b). Metagenomic approaches have shown a positive correlation between CTP (Child-Turcotte-Pugh) scores and the abundance of *Enterobacteriaceae* and *Veillonella*, whereas a negative correlation exists between CTP scores and *Bacteroidetes*. The metagenomic sequence data was assigned to the eggNOG database (evolutionary genealogy of genes: Non-supervised Orthologous Groups, http://eggnog.embl.de/) (Powell et al., 2014) and the KEGG database (Kyoto Encyclopedia of Genes and Genomes, Kyoto Encyclopedia of Genes and Genomics, http://www.genome.jp/kegg/) (Kanehisa et al., 2008). This was followed by statistical analysis of the relative abundance of genes assigned to each COG category (Clusters of Orthologous Groups of Proteins, http://www.ncbi.nlm.nih.gov/ COG) and KEGG metabolic pathway; furthermore, the functional and metabolic annotations of different genes were informed. Intestinal microbiota from cirrhotic patients contained a significant reduction in functional diversity compared with healthy patients, with an enrichment in material (amino acid, secondary metabolites, inorganic ion) transport and metabolism, energy production and conversion. In terms of metabolites, there was a distinct abundance of nitrogen, branched-chain amino acids, glutathione, and lipids, with a remarkable deficiency in the metabolites of bile acid and aromatic amino acid (Wei et al., 2013).

However, the function and metabolism predictions based on the metagenome sequencing have no explicit data support. Therefore, stool samples from subjects were collected and further efforts were performed by high-throughput sequencing of metaproteome and metabolome. The raw data of metaproteome sequencing has been uploaded to the proteomics database of iProx (Integrated Proteome Resources, http://www.iprox.org/my/index). A core-metaproteome database containing 119 proteins, specific to the gut microbiota of cirrhotic patients, was discovered. More than one third of the proteins from this database were assigned to material transport and metabolism; moreover, fourteen proteins had remarkably enhanced expression and seven proteins had unique expression in patients compared to the controls. In two metabolic pathways, which were map 00290 (valine, leucine and isoleucine biosynthesis) and map00770 (pantothenate and coenzyme A biosynthesis), the number of cirrhosis-unique proteins increased as the disease got worse, and in patients with a CTP score of C, the unique proteins almost coverd the entire metabolic pathway (Wei et al., 2016a).

In addition, metabolome sequencing on the gut microbiota showed significant separation in the composition of metabolites between the cirrhotic patients and the normal, illustrating remarkable increase in the abundance of amine, SCFAs (short-chain fatty acids), and unsaturated fatty acid, as well as decreased concentrations in both amino acid and sugar alcohol. Moreover, cirrhotic patients contained abnormal, and differential microbiota-metabolite correlations (Wei et al., 2016b).

## Cirrhosis-specific Metabolic Network in Gut

**Figure 1:**
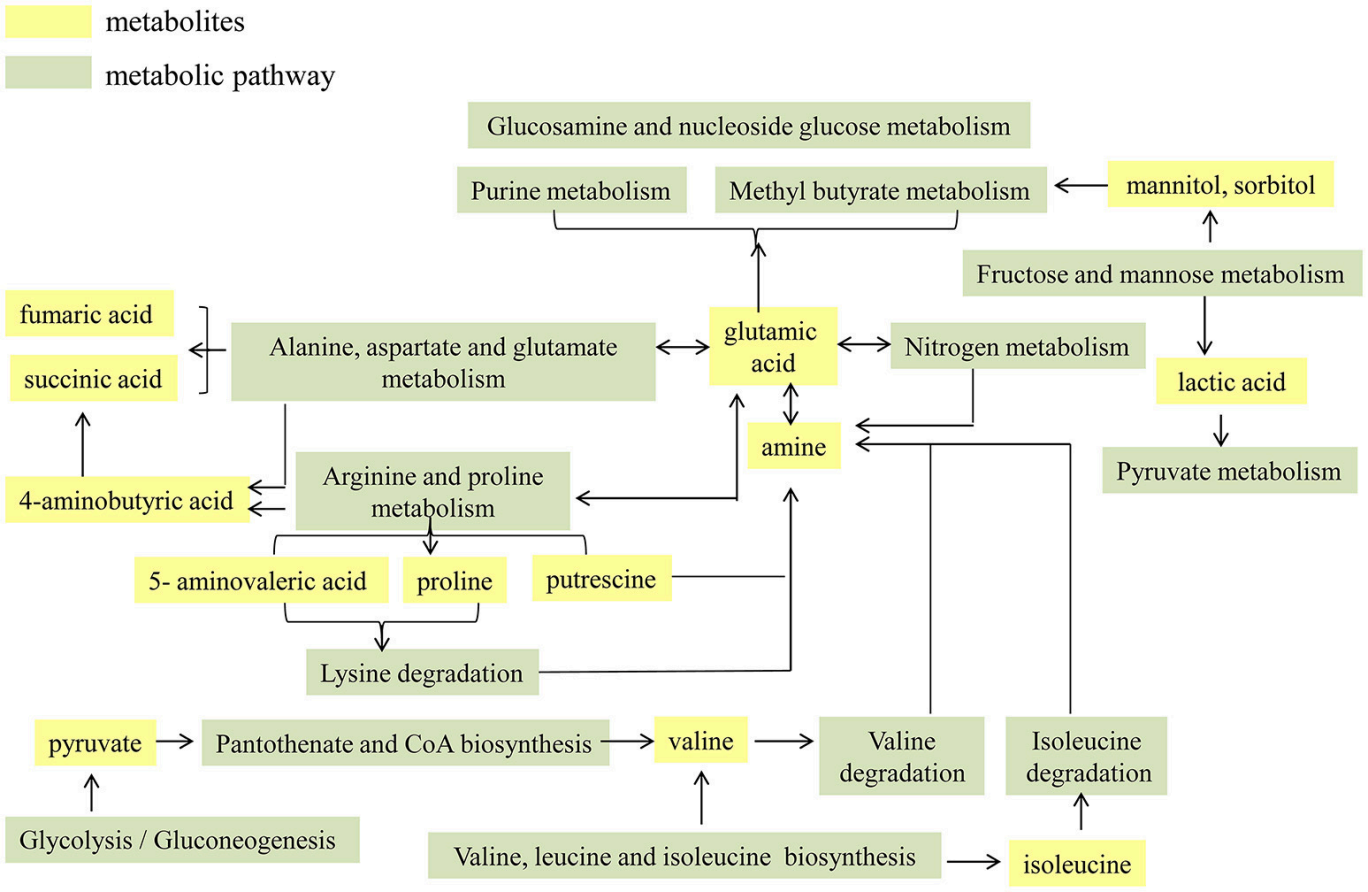
Cirrhosis-associated metabolic network of intestinal microbiota.

The functional proteins and metabolites that were of interest and abundantly detected were assigned to the original KEGG pathway database (http://www.kegg.jp/) (Kanehisa et al., 2008). As shown in Table 1, it was explicit that a total of seven metabolic pathways were significantly enhanced in cirrhotic patients, which were map00910: Nitrogen metabolism; map00250: Alanine, aspartate and glutamate metabolism; map00290: Valine, leucine and isoleucine biosynthesis; map00770: Pantothenate and CoA biosynthesis; map00010: Glycolysis/Gluconeogenesis; map00051: Fructose and mannose metabolism; and map00330: Arginine and proline metabolism. For an organism, the body's metabolic pathways are not independent but are rather interrelated. Therefore, in this study, the functional proteins and metabolites highly expressed in the patient's gut microbiota are considered as hubs, and the enhanced metabolic pathways are integrated to map the metabolic network of the cirrhosis-related gut microbiota (Figure 1). High levels of glutamate synthase, along with glutamate synthase in the intestine, in cirrhotic patients likely led directly to an elevation of glutamate, which further participates in the pathways of nitrogen, alanine, aspartate and glutamate metabolism. Enhancement in arginine and proline metabolism must have resulted in increased levels of proline and 5-aminopentanoate, both of which enter the lysine degradation pathway, in concert with enhanced nitrogen metabolism, resulting in more production of amines, further producing more ammonia. Increases in fructose and mannose metabolism must have resulted in more production of mannitol and sorbitol, both of which are further involved in the amino sugar and ribosomal glucose metabolism pathways. The biosynthesis of the valine and isoleucine pathways increases, and the glycolysis/gluconeogenic pathway enhances, resulting in more pyruvate accessing the pantothenate and coenzyme A biosynthesis pathways, which will also produce more valine. Degradation of valine and isoleucine produces more amines, further producing more ammonia.

**Table 1:** Summary of upregulated functional proteins and metabolites in cirrhotic patients.

**KEGG pathway**	**Proteins**	**Metabolites**
Nitrogen metabolism	Glutamate dehydrogenase	Glutamic acid
	Glutamine synthetase	
Alanine, aspartate, and glutamate metabolism	Glutamate dehydrogenase Glutamine synthetase	Fumaric acid, 4-aminobutyric acid, succinic acid, glutamic acid
Valine, leucine, and isoleucine biosynthesis	Ketol-acid reductoisomerase	Isoleucine, valine
	Dicarboxylic acid dehydrating enzyme	
Pantothenate and CoA biosynthesis	Ketol-acid reductoisomerase	Valine
	Dicarboxylic acid dehydrating enzyme	
Glycolysis/ Gluconeogenesis	Glyceraldehyde-3-phosphate dehydrogenase	Lactic acid
	Transaldolase	
	Phosphoglycerate kinase	
Fructose and mannose metabolism	XYLOSE isomerase	Lactic acid, mannitol, sorbitol
Arginine and proline metabolism	Glutamate dehydrogenase	Carbamide, 4-aminobutyric acid, 5-aminopylamine, glutamate, proline, hydroxyproline

## Discussion

There is a close anatomical and physiological association between the liver and the intestinal microbiota through the structure of the gut-liver axis. Intestinal microbiota dysbiosis is associated with the development of liver disease. Probiotics, prebiotics, synbiotics, and antibiotics can affect the host's health and ability to digest food through altering the composition of gut bacteria. Probiotics are microorganisms that confer a health benefit on the host when administered in adequate amounts. Probiotics mediate the intestinal microenvironment through four distinct mechanisms: (a) probiotics compete with pathogens for essential nutrients; (b) probiotics compete with pathogens for adhesion sites; (c) signaling of immune cells by probiotics can lead to the secretion of cytokines, thereby targeting the elimination of pathogens; and (d) probiotics can attack pathogenic organisms by releasing bacteriocins. Prebiotics are usually fiber compounds that are not easily digested by the human body and can be used as a substrate to stimulate the growth or activity of beneficial bacteria in the intestine. Fermentation is the main mechanism of action of beneficial bacteria using prebiotics in human gut. At present, research on prebiotics has mainly focused on the effects of prebiotics on *Bifidobacteria* and *Lactobacilli*, which have been recognized as beneficial intestinal bacteria and have beneficial effects on improving digestion and enhancing immune system function. Synbiotics are a combination of probiotics and prebiotics in a synergistic manner to improve the intestinal environment. Antibiotics alter the metabolic interactions of the gut microbiota by altering the number of gastrointestinal microbiota, and fully affect host metabolism, hormone levels, and immune homeostasis.

In a normal human gut, the microbiota participates in the degradation of amino acids from dietary intake, with the production of nitrogen-containing compounds, among which the most important nitrogenous waste product is ammonia. The N-containing compounds are transported to the liver through the portal vein, where more than 80% are metabolized through the urea cycle pathway and/or excreted immediately (Bajaj, 2010). The initial equilibrium state of the intestinal microbiota structure, function and metabolism was broken due to the liver cirrhosis. The abnormal intestinal microbiota's ecological environment outstandingly embodied the enhanced amino acid metabolism with an excessive production of ammonia. The liver cells were functionally damaged and incapable of metabolizing the waste products effectively. The redundant ammonia entered the blood circulation and crossed the blood-brain barrier, further exploited by astrocyte to synthesize glutamine from glutamate. The surge in the level of glutamine causes brain edema, further increasing the risk in developing hepatic encephalopathy (Bajaj, 2010). It was reported that near 40% or more of cirrhotic patients suffer from hepatic encephalopathy (Borkakoty et al., 2017).

This study summarized the cirrhosis-specific metabolic network of intestinal microbiota, illustrating that the abnormal gut microbiota metabolic pathway resulted in the excessive production of ammonia, which could not be eliminated completely by the damaged liver and crossed the blood-brain barrier, and might facilitate the acquisition of hepatic encephalopathy. In the future, many objects will be recruited to further verify and improve the cirrhosis-specific metabolic network of intestinal microbiota in this study. Other than this, an intestinal micro-ecological treatment method related to liver cirrhosis needs to be formulated according to the improved metabolic pathway. Consequently, actively regulating the balance of the intestinal microbiota community and metabolism will have a significant effect on preventing the deterioration and improving the prognosis of the disease. Our research will provide guiding significance for cirrhosis clinically in the future.

## Author Contributions

JZ and XW designed research. XW, JZ, XJ, XZ performed research. HL, WL, and RF contributed analytic tools. XW, JZ, XJ, XZ, and HL analyzed data. XW, JZ, XJ and JY wrote the paper.

### Conflict of Interest Statement

The authors declare that the research was conducted in the absence of any commercial or financial relationships that could be construed as a potential conflict of interest.

## Abbreviations

CTP: Child-Turcotte-Pugh
eggNOG: evolutionary genealogy of genes: Non-supervised Orthologous Groups
COG: Clusters of Orthologous Groups
SCFAs: short-chain fatty acids.
